# PD-1^+^ IFN-γ^+^ subset of CD8^+^ T cell in circulation predicts response to anti–PD-1 therapy in NSCLC

**DOI:** 10.3389/fonc.2023.1182301

**Published:** 2023-06-13

**Authors:** Wenxiu Chen, Yiting Hua, Conghui Shan, Jia Wei, Yutong Zhou, Shiyang Pan

**Affiliations:** ^1^ Department of Laboratory Medicine, The First Affiliated Hospital of Nanjing Medical University, Nanjing, China; ^2^ Branch of National Clinical Research Center for Laboratory Medicine, Nanjing, China

**Keywords:** PD-1, IFN-γ, NSCLC, circulation, chemotherapy

## Abstract

**Background:**

Treatment with programmed cell death protein-1 (PD-1) antibodies has minimal response rates in patients with non–small cell lung cancer (NSCLC), and, actually, they are treated with chemotherapy combined with anti–PD-1 therapy clinically. Reliable markers based on circulating immune cell subsets to predict curative effect are still scarce.

**Methods:**

We included 30 patients with NSCLC treated with nivolumab or atezolizumab plus platinum drugs between 2021 and 2022. Whole blood was collected at baseline (before treatment with nivolumab or atezolizumab). The percentage of circulating PD-1^+^ Interferon-γ (IFN-γ^+^) subset of CD8^+^ T cell was determined by flow cytometry. The proportion of PD-1^+^ IFN-γ^+^ was calculated after gating on CD8^+^ T cells. Neutrophil/lymphocyte ratio (NLR), relative eosinophil count (%), and Lactate dehydrogenase (LDH) concentration at baseline of included patients were extracted from electronic medical records.

**Results:**

The percentage of circulating PD-1^+^ IFN-γ^+^ subset of CD8^+^ T cell at baseline in responders was significantly higher than those in non-responders (P < 0.05). Relative eosinophil count (%) and LDH concentration in responders showed no significance between non-responders and responders. NLR in responders was significantly lower than those in non-responders (*P* < 0.05). Receiver operation characteristic (ROC) analysis found that the areas under the ROC curve for PD-1^+^ IFN-γ^+^ subset of CD8^+^ T cell and NLR were 0.7781 (95% CI, 0.5937–0.9526) and 0.7315 (95% CI, 0.5169–0.9461). Moreover, high percentage of PD-1^+^ IFN-γ^+^ subset in CD8^+^ T cells was relevant to long progression-free survival in patients with NSCLC treated with chemotherapy combined with anti–PD-1 therapy.

**Conclusion:**

The percentage of circulating PD-1^+^ IFN-γ^+^ subset of CD8^+^ T cell could be a potential marker at baseline to predict early response or progression in patients with NSCLC receiving chemotherapy combined with anti–PD-1 therapy.

## Introduction

Non–small cell lung cancer (NSCLC) is currently one of the leading causes of morbidity and mortality in the world (1), and, despite some progress in the conventional treatment of NSCLC, such as surgery, radiotherapy, chemotherapy, and targeted therapy, the 5-year survival rate of patients with lung cancer is still low (2). Immune checkpoint molecules such as programmed cell death protein-1/programmed death-ligand 1 (PD-1/PD-L1) blockade therapy offer a new model for NSCLC treatment, but only about 30% of patients with NSCLC benefit from PD-1/PD-L1 blockade therapy (3). Biomarkers used to predict response include tumor burden, PD-1 expression balance between effector and regulatory T cells in tumor microenvironment, PD-L1 expression by the tumor cells, and composition of the gut microbiota (4–7). Most of these predictive biomarkers are closely related to activation and function of CD8^+^ T cells in the tumor microenvironment, confirming their pivotal role for prediction and evaluation of immunotherapy efficacy (8, 9). However, the predictive value of CD8^+^ T cells in circulation remains to be elucidated. It is worth noting that studies have found the proliferation of PD-1^+^ CD8^+^ T cells in peripheral blood after PD-1–targeted therapy in patients with lung cancer (10).

Unlike tumor-infiltrating CD8^+^ T-lymphocyte populations, which consist mainly of high levels of PD-1, CD8^+^ T-lymphocyte populations in circulation mainly express intermediate or low levels of PD-1 (11). Furthermore, tumor-infiltrating CD8^+^ T cells expressing high levels of PD-1 are impaired in classical effector cytokine production and show limited response to PD-1 blockade therapy (12). Classical effector cytokines including IFN-γ may be used to evaluate the functional status of CD8^+^ T cells expressing low PD-1 expression in peripheral circulation, thus predicting the therapeutic effect of anti–PD-1 therapy. In daily practice, only a few patients with NSCLC treated with anti–PD-1 therapy alone and most patients were treated with chemotherapy combined with anti–PD-1 therapy. In this study, we investigated whether the percentage of circulating PD-1^+^ IFN-γ^+^ CD8^+^ T cell at baseline could be a potential marker to predict early response or progression in patients with NSCLC receiving chemotherapy combined with anti–PD-1 therapy.

## Methods

### Patients

A total of 30 patients with advanced NSCLC treated with nivolumab or atezolizumab plus platinum drugs were eligible for this study. All patients included met the following inclusion criteria: ≥18 years of age; histologically or pathologically confirmed stage III or stage IV or recurrent NSCLC; and treated with platinum in anterior lines and treated with nivolumab or atezolizumab plus platinum drugs in the follow-up treatment. The exclusion criteria include the following: nivolumab or atezolizumab was stopped during treatment due to immune-related adverse events; and follow-up was less than 12 months. Clinical features were extracted from electronic patient records, and the study was approved by the Ethics Committee of the First Affiliated Hospital of Nanjing Medical University (Nanjing, China) (No. 2022-SR-695), and informed consent was taken from all the patients.

### Sample collection and detection

Peripheral blood samples were obtained at baseline (before treatment with nivolumab or atezolizumab). The percentage of circulating PD-1^+^ IFN-γ^+^ CD8^+^ T cell was determined by flow cytometry.

### Response assessment

Response was evaluated by computed tomography (CT) for the first visit to the clinic. Responders were defined as complete response, partial response, or stable disease ≥ 6 months. Non-responders were defined as stable disease < 6 months or progressive disease according to RECISTv1.1 (The Response Evaluation Criteria In Solid Tumors).

### Isolation of peripheral blood mononuclear cells

Peripheral blood mononuclear cells (PBMCs) were isolated by using Ficoll-Hypaque density gradient. Then, PBMCs were gained from the Ficoll/serum interface by density gradient centrifugation (2,000*g*, 20 min) and washed twice with phosphate-buffered saline (PBS).

### Flow cytometry analysis

Cells were stained with Fluorescein isothiocyanate (FITC)-conjugated CD8 (BD Biosciences, USA) and Allophycocyanin (APC) -conjugated CD279 (Biosciences (BD), USA) antibodies for 20 min according to the manufacturer’s instructions. Next, cells were fixed and permeabilized for 20 min using Cytofix/Cytoperm reagent (BD Biosciences) and subsequently stained with antibodies to intracellular molecule Phycoerythrin (PE)-conjugated IFN-γ. For intracellular cytokine IFN-γ staining, the cells were stimulated for 4 h with the Cell Stimulation and Protein Transport Inhibitor Cocktail (Biogems) before staining.

### Verifying the combined performance of PDCD1 and IFNG in predicting patient prognosis

Gene expression information and corresponding clinicopathological data were downloaded from The Cancer Genome Atlas (TCGA) database. We obtained 491 lung squamous cell carcinoma (LUSC) and 503 lung adenocarcinoma (LUAD). R (version 4.1.0) was utilized to process and analyze the data. Survival curves could be acquired through the R package “survival” and “survminer”.

### Statistical analysis

All statistical evaluations were performed using GraphPad Prism, version 7.0 (GraphPad Software, Inc., SigmaPlot). Student’s t-test was used for normally distributed data, and Mann–Whitney test was used for skewed distributed data statistical analysis of group differences. Differences were considered statistically significant when *p* < 0.05. Multiple comparisons between non-responders and responders use false discovery rate (FDR) correction. Only significant *p*-values are displayed. Receiver operating characteristic (ROC) curve and area under the ROC curve (AUC) were used to compare the discrimination capacity for each of circulating PD-1^+^ IFN-γ^+^ CD8^+^ T cells, neutrophil/lymphocyte ratio (NLR), relative eosinophil count (%), and LDH concentration. The patients were divided into high- and low-expression groups according to the cutoff point value of the percentage of circulating PD-1^+^ IFN-γ^+^ CD8^+^ T cell. Survival analysis were carried out by the Kaplan–Meier method. Univariable analysis and multivariable logistic regression analysis were performed to identify hazard ratios (HRs) for progression-free survival (PFS) by grouping median value of each variable.

## Results

### Patient characteristics

A total of 30 patients were enrolled in this study between April 2021 and December 2021. Enrolled patients were followed up for 12 months. Whole-blood samples for baseline were available of 30 patients. The patient characteristics are listed in **Table 1**. All 30 patients had at least one CT scan from electronic patient records. We use first CT scan as early tumor response evaluation result.

**Table 1:** Patients’ demographic and tumor characteristics.

Clinicopathological features	Peripheral blood samples, n = 30
Sex, male/female	19/11
Age in years	47–81, median (67)
Metastatic status, yes/no	10/20
Smoking status, yes/no	18/12
Histological type, adenocarcinoma/squamous cell carcinoma	14/16

### Increased PD-1 expression is linked to IFN-γ expression of circulating CD8^+^ T cell

**Figure 1:**
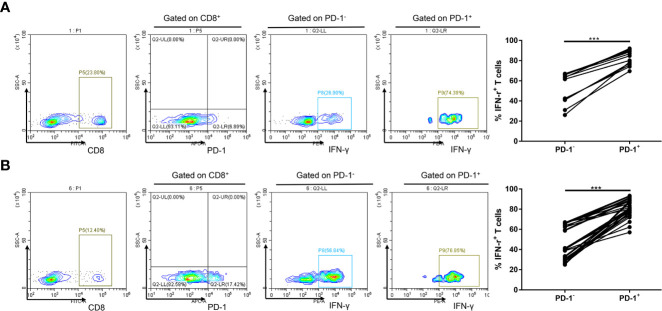
Increased PD-1 expression is linked to IFN-γ expression of CD8^+^ T cell in circulation. **(A)** A representative flow cytometry and analysis of IFN-γ^+^ cell percentages in PD-1^+^ and PD-1^−^ subsets of CD8^+^ T lymphocytes in circulation of 12 healthy controls. **(B)** A representative flow cytometry and analysis of IFN-γ^+^ cell percentages in PD-1^+^ and PD-1^−^ subsets of CD8^+^ T lymphocytes in circulation of 30 patients with NSCLC. ***P < 0.001.

Exhausted CD8^+^ T cells have decreased IFN-γ production, in part, due to overexpression of inhibitory receptors such as PD-1 (13). Considering the low level of PD-1 expression in peripheral blood, we investigated whether increased PD-1 expression could influence CD8^+^ T-cell effector function in circulation. Notably, we observed that the percentages of IFN-γ–producing CD8^+^ PD-1^+^ T cells in circulation were significantly higher than those in CD8^+^ PD-1^−^ T cells in healthy controls (*P* < 0.001; **Figure 1A**) and in patients with NSCLC (*P* < 0.001; **Figure 1B**). PD-1 is expressed on activated T, natural killer and B lymphocytes, macrophages, dendritic cells, and monocytes (13, 14), and expression of PD-1 in peripheral blood CD8^+^ T cells may indicate an activated state instead of exhaustion (14). These data demonstrate that circulating CD8^+^ PD-1^+^ T cells are inclined to produce IFN-γ.

### PD-1^+^ IFN-γ^+^ cell percentages in CD8^+^ T cells in circulation predict therapeutic response to anti–PD-1 in patients with NSCLC

**Figure 2:**
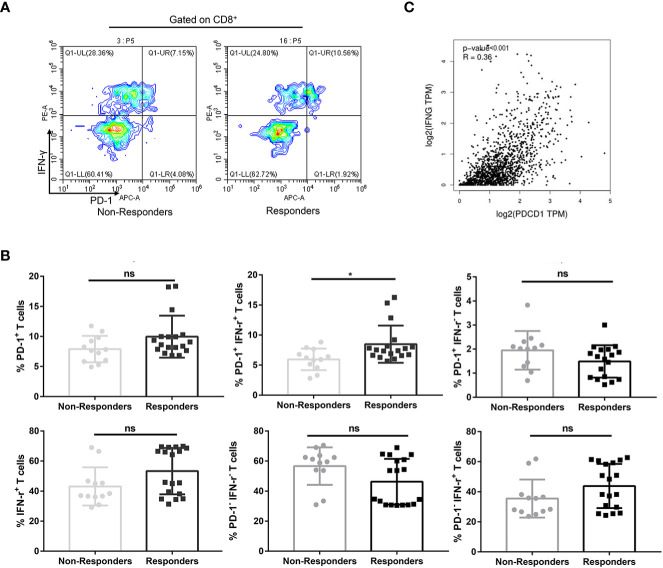
Percentages of PD-1^+^ IFN-γ^+^ cell of CD8^+^ T cells in circulation predicted therapeutic response to anti–PD-1 therapy in combination with chemotherapy in patients with NSCLC. **(A)** A representative flow cytometry analysis of PD-1^+^ IFN-γ^+^ cell percentages of CD8^+^ T cells in circulation of non-responders (n = 12) and responders (n = 18). **(B)** Statistical analysis of PD-1^+^, IFN-γ^+^, PD-1^+^ IFN-γ^+^, PD-1^−^ IFN-γ^−^, PD-1^+^ IFN-γ^−^, and PD-1^−^ IFN-γ^+^ subsets of CD8^+^ T lymphocytes in circulation of non-responders (n = 12) and responders (n = 18). **(C)** Association of PDCD1 expression with IFNG expression in NSCLC calculated by GEPIA2 using TCGA in NSCLC. No significant (ns), *P < 0.05.

Exhausted CD8^+^ T cells may not be reinvigorated by anti–PD-1 therapy, referred to as non-responders (15). Having found that circulating CD8^+^ PD-1^+^ T cells are inclined to produce IFN-γ, we further investigated the implications of PD-1^+^ IFN-γ^+^ cell percentages in CD8^+^ T cells in prediction of benefit of anti–PD-1 therapy. PD-1^+^, IFN-γ^+^, PD-1^+^ IFN-γ^−^, PD-1^−^ IFN-γ^+^, and PD-1^−^ IFN-γ^−^ subsets of CD8^+^ T lymphocytes in non-responders were similar to those in responders (*P* > 0.05, FDR-corrected *P >* 0.05; **Figure 2B**). However, PD-1^+^ IFN-γ^+^ subset in CD8^+^ T cells in responders was significantly higher than those in non-responders (*P* < 0.05, FDR-corrected *P <* 0.05; **Figures 2A, B**), suggesting that IFN-γ–producing CD8^+^ PD-1^+^ T cells in circulation are hallmarks of T-cell viability and may benefit from anti–PD-1 therapy. Meanwhile, Gene Expression Profiling Interactive Analysis, version 2 (GEPIA2) calculations based on TCGA database show that programmed cell death 1 (PDCD1) expressions are associated with interferon gamma (IFNG) expression in NSCLC (**Figure 2C**).

### PD-1^+^ IFN-γ^+^ cell percentages in CD8^+^ T cells in circulation are associated with progression-free survival in patients with NSCLC treated with anti–PD-1 therapy

**Figure 3:**
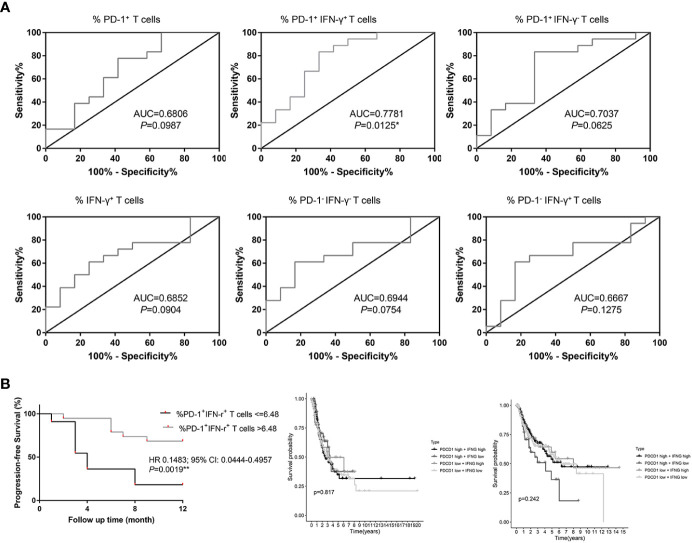
Percentages of PD-1^+^ IFN-γ^+^ cell of CD8^+^ T cells in circulation are associated with progression-free survival (PFS) in patients with NSCLC treated with anti–PD-1 therapy in combination with chemotherapy. **(A)** ROC curves for the correlation of PD-1^+^, IFN-γ^+^, PD-1^+^ IFN-γ^+^, PD-1^−^ IFN-γ^−^, PD-1^+^ IFN-γ^−^, and PD-1^−^ IFN-γ^+^ subsets of CD8^+^ T lymphocytes in circulation with response (non-responders and responders) to treatment in patients with NSCLC. **(B)** PFS in patients with NSCLC according to cutoff point value of PD-1^+^ IFN-γ^+^ cells of circulating CD8^+^ T cells (left) and PFS in the lung adenocarcinoma (middle) and lung squamous cell carcinoma (right) patients in TCGA cohort according to cutoff point value of PDCD1 and IFNG transcriptome data. *P < 0.05; **P < 0.01.

PD-1^+^ IFN-γ^+^ cell percentages of CD8^+^ T cells were significantly associated with response to chemotherapy combined with anti–PD-1 therapy [median level (%): responders, 7.46% (Q1–Q3: 6.61% to 8.42%); non-responders, 6.06% (Q1–Q3: 4.72%–7.43%); *P* = 0.0116, FDR-corrected *P* = 0.0116], with an AUC in the ROC function of 0.7781 (95% CI, 0.5937–0.9526) (*P* = 0.0125; **Figure 3A**). We also plotted ROC curves for several other groups. PD-1^+^ cell percentages of CD8^+^ T cells [median level (%): responders, 8.84% (Q1–Q3: 7.81% to 10.05%); non-responders, 7.74% (Q1–Q3: 5.88%–9.66%); *P* = 0.1017, FDR-corrected *P =* 0.3051], with an AUC in the ROC function of 0.6806 (95% CI, 0.4766–0.8845) (*P* = 0.0987; **Figure 3A**), IFN-γ^+^ cell percentages of CD8^+^ T cells [median level (%): responders, 55.06% (Q1–Q3: 37.37% to 68.76%); non-responders, 37.73% (Q1–Q3: 36.26%–46.32%); *P* = 0.0949, FDR-corrected *P =* 0.2135], with an AUC in the ROC function of 0.6852 (95% CI, 0.4924–0.8779) (**Figure 3A**, *P* = 0.0904); PD-1^−^ IFN-γ^−^ cell percentages of CD8^+^ T cells [median level (%): responders 44.15% (Q1–Q3: 30.86% to 61.75%); non-responders 60.34% (Q1–Q3: 53.77%–63.41%); *P* = 0.0772, FDR-corrected *P =* 0.1389], with an AUC in the ROC function of 0.6944 (95% CI, 0.5041–0.8848) (*P* = 0.0754; **Figure 3A**); PD-1^+^ IFN-γ^−^ cell percentages of CD8^+^ T cells [median level (%): responders, 1.63% (Q1–Q3: 0.80% to 1.97%); non-responders, 2.03% (Q1–Q3: 1.32%–2.27%); *P* = 0.0649, FDR-corrected *P =* 0.0834], with an AUC in the ROC function of 0.7037 (95% CI, 0.5025–0.9049) (*P* = 0.0625; **Figure 3A**); and PD-1^−^ IFN-γ^+^ cell percentages of CD8^+^ T cells [median level (%): responders, 43.31% (Q1–Q3: 28.77% to 59.56%); non-responders, 32.02% (Q1–Q3: 26.79%–37.73%); *P* = 0.1345, FDR-corrected *P =* 0.6052], with an AUC in the ROC function of 0.6667 (95% CI, 0.4629–0.8704) (*P* = 0.1275; **Figure 3A**) did not show significant difference between responders and non-responders. Using the ROC curve to determine the value of PD-1^+^ IFN-γ^+^ cell percentages of CD8^+^ T cells to predict response, we chose >6.48% as the cutoff point that combined maximal sensitivity (83.33%; 95% CI, 58.58%–96.42%) with best specificity (66.67%; 95% CI, 34.89%–90.08%). Therefore, we use cutoff point value of PD-1^+^ IFN-γ^+^ cell percentages of CD8^+^ T cells in 30 patients with NSCLC to calculate PFS (time from the time starting anti–PD-1 therapy until disease progression or death from any cause, follow-up for 12 months). PD-1^+^ IFN-γ^+^ cell percentages of CD8^+^ T cells in responders and non-responders are listed in **Table 2**. We found that patients with PD-1^+^ IFN-γ^+^ cell percentages of CD8^+^ T cells >6.48% had a significant longer PFS compared with patients with PD-1^+^ IFN-γ^+^ cell percentages of CD8^+^ T cells ≤6.48% (*P* < 0.01; **Figure 3B**, left). These data demonstrate that circulating PD-1^+^ IFN-γ^+^ cell percentages of CD8^+^ T cells may serve as markers of NSCLC treated with chemotherapy combined with anti–PD-1 therapy to predict progression. However, transcriptome data from TCGA in LUAD (**Figure 3B**, middle) and LUSC (**Figure 3B**, right) showed that PFS was not significantly associated with PDCD1and IFNG expression levels stratified at median value by ifelse function in R. The contradictory results may be due to the short follow-up time of our study and the insufficient number of included cases.

**Table 2:** Response at first CT scan stratified at the level of PD-1^+^IFN-γ^+^ in CD8^+^ T cells at baseline.

Response	PD-1^+^ IFN-γ^+^ T cells ≤ 6.48%	PD-1^+^ IFN-γ^+^ T cells > 6.48%
	11	19
Responders	3 (27.3)	15 (78.9)
Non-Responders	8 (72.7)	4 (21.1)

### Comparison of other blood-based biomarkers and PD-1^+^ IFN-γ^+^ cell percentages of CD8^+^ T cells in the predictive and prognostic effect of anti–PD-1 therapy

**Figure 4:**
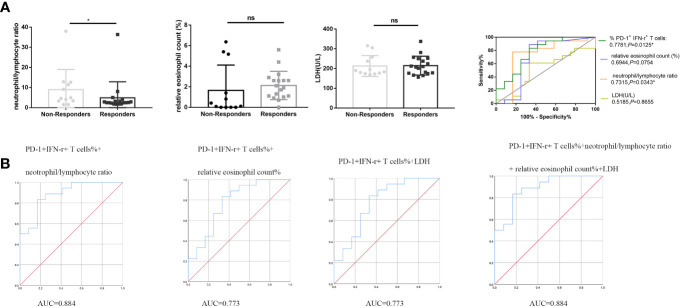
PD-1^+^ IFN-γ^+^ cell percentages of CD8^+^ T cells, NLR, relative eosinophil count (%), and LDH concentration in the predictive and prognostic effect of anti–PD-1 therapy. **(A)** Statistical analysis of NLR, relative eosinophil count (%), and LDH concentration in circulation of non-responders (n = 12) and responders (n = 18) and ROC analysis. **(B)** Combined predictive efficiency ROC analysis of PD-1^+^ IFN-γ^+^ cell percentages of CD8^+^ T cells and NLR and relative eosinophil count (%) and LDH concentration. *P < 0.05; ns, *P* > 0.05.

Blood indicators that have been reported to be related to the efficacy of anti–PD-1 therapy include NLR, relative eosinophil count (%), and LDH concentration (16). Baseline NLR, relative eosinophil count (%), and LDH concentration values for enrolled patients were obtained from the electronic medical record system. Statistical analysis showed no significant difference in relative eosinophil count (%) and LDH concentration between responders and non-responders (*P* > 0.05; **Figure 4A**). NLR in responders was significantly lower than those in non-responders (*P* < 0.05; **Figure 4A**). ROC curve analysis was performed to evaluate whether baseline NLR, relative eosinophil count (%), and LDH concentration could predict the efficacy of anti–PD-1 therapy: NLR [median level (%): responders, 2.55% (Q1–Q3: 2.17% to 3.48%); non-responders, 4.78% (Q1–Q3: 3.32%–12.36%); *P* = 0.0339, FDR-corrected *P =* 0.0381], with an AUC in the ROC function of 0.7315 (95% CI, 0.5169–0.9461) (*P* = 0.0343; **Figure 4A**, right); relative eosinophil count (%) [median level (%): responders, 2.00% (Q1–Q3: 1.07% to 2.67%); non-responders, 0.26% (Q1–Q3: 0%–4.23%); *P* = 0.0761, FDR-corrected *P =* 0.1141], with an AUC in the ROC function of 0.6944 (95% CI, 0.4614–0.9275) (*P* = 0.0754; **Figure 4A**, right); and LDH concentration [median level (%): responders, 208 (Q1–Q3: 175 to 250); non-responders, 186 (Q1–Q3: 174–252); *P* = 0.8758, FDR-corrected *P* > 0.99], with an AUC in the ROC function of 0.5185 (95% CI, 0.2996–0.7374) (*P* = 0.8655; **Figure 4A**, right). Although NLR can be used to distinguish responders from non-responders in patients with NSCLC treated with anti–PD-1 therapy as reported in the literature, its AUC is not as good as that of PD-1^+^ IFN-γ^+^ cell percentages of CD8^+^ T cells. Therefore, we use ROC curve to analyze the predictive efficacy of PD-1^+^ IFN-γ^+^ cell percentages of CD8^+^ T cells combined with NLR, relative eosinophil count (%), and LDH concentration. As shown in **Figure 4B**, the combined detection of PD-1^+^ IFN-γ^+^ cell percentages of CD8^+^ T cells and NLR improved the predictive efficiency (AUC = 0.884; 95% CI, 0.763–1.000); the combined detection of PD-1^+^ IFN-γ^+^ cell percentages of CD8^+^ T cells and relative eosinophil count (%)/LDH concentration improved the predictive efficiency (AUC = 0.773; 95% CI, 0.594–0.953); and the combined detection of PD-1^+^ IFN-γ^+^ cell percentages of CD8^+^ T cells and relative eosinophil count (%) and LDH concentration improved the predictive efficiency (AUC = 0.884; 95% CI, 0.763–1.000).

Our previous data showed that PD-1^+^ IFN-γ^+^ cell percentages of CD8^+^ T cells were associated with PFS. We divided the included patients into two groups according to median value of PD-1^+^ IFN-γ^+^ cell percentages of CD8^+^ T cells, NLR, relative eosinophil count (%), and LDH concentration. The significant relationship between PD-1^+^ IFN-γ^+^ cell percentages of CD8^+^ T cells and PFS was observed both in univariable analysis and multivariable logistic regression analysis. Detailed group information is shown in **Table 3**. These data suggest that PD-1^+^ IFN-γ^+^ cell percentages of CD8^+^ T cells may be an independent prognostic factor for anti–PD-1 therapy, superior to the currently reported markers represented by NLR, relative eosinophil count (%), and LDH concentration.

**Table 3:** Logistic regression analysis between serum biomarkers and PFS in patients with NSCLC with anti–PD-1 therapy and chemotherapy.

Characteristics	Univariable analysis		Multivariate analysis	
	HR (95% CI)	*P*	HR (95% CI)	*P*
Sex (male/female)	0.84 (0.29–2.36)	0.736		
Age (≤67 *vs*. >67)	0.73 (0.27–2.02)	0.549		
Smoking (no/yes)	0.66 (0.21–2.07)	0.473		
Histological (adenocarcinoma/squamous)	0.71 (0.25–2.00)	0.518		
Metastatic (no/yes)	0.32 (0.11–0.87)	0.027*	0.44 (0.15–1.29)	0.138
PD-1^+^ IFN-γ^+^ T cells (≤6.83% *vs*. >6.83%) NLR (≤3.21% *vs*. >3.21%)	4.05 (1.27–12.89) 0.38 (0.13–1.11)	0.018* 0.076	3.12 (1.04–10.27) 0.35 (0.11–1.06)	0.048* 0.064
Eosinophil% (≤1.35% *vs*. >1.35%) LDH (≤198 *vs*. >198)	2.53 (0.86–7.43) 0.39 (0.13–1.15)	0.091 0.087	1.92 (0.62–5.93) 0.61 (0.19–1.84)	0.256 0.374

*P<0.05.

## Discussion

Platinum-based chemotherapy remains the standard treatment for most patients with NSCLC (17). Despite the emergence of various platinum compounds, the therapeutic effect is still not up to expectations. Nevertheless, the treatment landscape for NSCLC has changed rapidly. Immune checkpoint inhibitor such as anti–PD-1 therapy combined with or without chemotherapy as first-line therapy has been widely studied (18–20). Dafni et al. have demonstrated that the combination of pembrolizumab plus chemotherapy and atezolizumab plus chemotherapy shows a better curative effect than pembrolizumab monotherapy and chemotherapy-monotherapy in their cohort (21). During our study design, it was also found that few patients were clinically treated with immunotherapy alone, so our study included patients who used nivolumab or atezolizumab plus platinum drugs. However, only approximately 30% of patients experience long-term benefit from anti–PD-1 therapy (3) plus chemotherapy, and the cost is high. Predictive biomarkers for clinical responses remain urgent. Existing predictive markers, including the expression of PD-L1 in tumor cells (22), tumor mutational burden, and gene mutation repair ability (23), and the expression level of PD-1 in tumor immune infiltrating CD8^+^ T cells (7) have limited value. Liquid immune profile–based signature for predicting response of anti–PD-1 therapy is favored by researchers as easy to obtain and non-invasive markers.

In this study, we demonstrate that PD-1^+^ IFN-γ^+^ subset of CD8^+^ T cells in the peripheral blood at baseline can predict response and PFS to anti–PD-1 therapy combined with chemotherapy in patients with NSCLC. Kamphorst et al. found proliferation of CD8^+^ T cells in peripheral blood of patients with NSCLC after PD-1–targeted therapy (10), suggesting that low or median PD-1 level of CD8^+^ T cells in peripheral blood may indicate activation of CD8^+^ T cells rather than exhaustion like in tumor microenvironment and is related to the efficacy of PD-1 immunotherapy. IFN-γ secretion of CD8^+^ T cells represents the activity and anti-tumor ability; thus, patients with high PD-1^+^ IFN-γ^+^ cell percentages of CD8^+^ T cells at baseline tend to benefit from PD-1 therapy.

Some limitations remain in this study. We did not collect blood samples during anti–PD-1 therapy plus chemotherapy after the treatment initiated and thus not be able to measure the effect of this marker to monitor and evaluate patients treated with anti–PD-1 therapy with chemotherapy. The first CT assessment used to identify early responders and non-responders may not distinguish between pseudoprogression and progression (24–26). Moreover, we did not include patients with chemotherapy-monotherapy or nivolumab/atezolizumab monotherapy to compare PD-1^+^ IFN-γ^+^ cell percentages of CD8^+^ T cells because there were few patients with immunotherapy alone enough to be included in our study. In addition, only 30 patient samples were included in our study, and the follow-up time is relatively short; more patients will be enrolled in the future to further verify our conclusions.

In conclusion, we have found that a new PD-1^+^ IFN-γ^+^ subset of CD8^+^ T cells can predict responses in patients with NSCLC during treatment with nivolumab or atezolizumab plus platinum. Furthermore, PD-1^+^ IFN-γ^+^ cell percentages of CD8^+^ T cells at baseline correlate with PFS in patients with NSCLC and may be an independent prognostic factor for anti–PD-1 therapy. PD-1^+^ IFN-γ^+^ cell percentages of CD8^+^ T cells in circulation are superior to the currently reported serum markers represented by NLR, relative eosinophil count (%), and LDH concentration in predictive and prognostic value of anti–PD-1 therapy. All these data suggest that, following confirmation and extension of our results in future, peripheral blood immune cell biomarkers may provide a precious and practical strategy to predict early responses to anti–PD-1 therapy in combination with chemotherapy that may assist in the treatment of patients with NSCLC.

## Data availability statement

The raw data supporting the conclusions of this article will be made available by the authors, without undue reservation.

## Ethics statement

The studies involving human participants were reviewed and approved by Institutional Ethics Committee of the First Affiliated Hospital of Nanjing Medical University. The patients/participants provided their written informed consent to participate in this study.

## Author contributions

WC performed the literature search and data analysis and wrote the manuscript. YH wrote part of the manuscript and polished the manuscript. CS, JW and YZ revised the manuscript. SP designed and supervised the study. All authors contributed to the article and approved the submitted version.
